# USING SERIAL TRICHOTOMIZATION WITH NEUROPSYCH MEASURES TO INFORM DECISIONS ON FITNESS TO DRIVE AMONG OLDER ADULTS

**DOI:** 10.1093/geroni/igz038.2881

**Published:** 2019-11-08

**Authors:** Stephanie Yamin, Valerie Ranger, Arne Stinchcombe, Frank Knoefel, Michel Bédard

**Affiliations:** 1 Saint Paul University (Ottawa), Ottawa, Ontario, Canada; 2 Bruyere Continuing Care, Ottawa, Ontario, Canada; 3 Lakehead University, Thunder Bay, Ontario, Canada

## Abstract

Older adults report that driving provides a sense of independence and wellbeing. For some older adults, driving cessation becomes necessary due to their health status having an impact on their ability to drive safely. Decisions related to driving cessation are difficult and often left to the clinical judgement of primary care physicians. There is an interest in developing a method that could help assist physicians in making that determination. To date, there is no neuropsychological test that produces an acceptable level of sensitivity and specificity allowing for the determination of an individual’s fitness to drive. Serial trichotomization involves classifying drivers as either pass, fail or indeterminate based on cut-points that leads to 100% sensitivity and specificity. The purpose of this study was to examine the serial trichotomization method using four common neuropsychological tests (i.e., 3MS, Trails A & B, clock drawing). Sensitivity and specificity for each test were established using a medical expert’s clinical judgement. Charts of 105 patients at a tertiary memory disorders clinic were reviewed and data related to neuropsychological test scores and clinical judgement around fitness to drive were abstracted. After applying the trichotomization, 38.1% of the sample were classified as unfit to drive, 36.1% were classified as indeterminate, and 25.8% were classified as fit to drive. This study adds to the growing body of literature supporting the use of serial trichotomization to streamline decision-making about fitness to drive.

